# Fabrication of pH/Reduction Sensitive Polyethylene Glycol-Based Micelles for Enhanced Intracellular Drug Release

**DOI:** 10.3390/pharmaceutics13091464

**Published:** 2021-09-14

**Authors:** Yang Yang, Fuwei Yang, Xiaotian Shan, Jiamin Xu, Wenjie Fang, Juan Zhou, Lipeng Qiu, Jinghua Chen

**Affiliations:** Department of Pharmaceutics, School of Pharmaceutical Sciences, Jiangnan University, Wuxi 214122, China; yangy@jiangnan.edu.cn (Y.Y.); 7181505005@stu.jiangnan.edu.cn (F.Y.); 18861824852@163.com (X.S.); 6191504015@stu.jiangnan.edu.cn (J.X.); 6191502024@stu.jiangnan.edu.cn (W.F.); juanzhou@jiangnan.edu.cn (J.Z.)

**Keywords:** polymeric micelles, doxorubicin, pH/reduction sensitive release, antitumor effect

## Abstract

At present, the drug is still difficult to release completely and quickly only with single stimulation. In order to promote the rapid release of polymeric micelles at tumor site, pH/reduction sensitive polymers (PCT) containing disulfide bonds and orthoester groups were synthesized. The PCT polymers can self-assemble in water and entrap doxorubicin to form drug-loaded micelles (DOX/PCT). In an in vitro drug release experiment, the cumulative release of DOX/PCT micelles in the simulated tumor microenvironment (pH 5.0 with GSH) reached (89.7 ± 11.7)% at 72 h, while it was only (16.7 ± 6.1)% in the normal physiological environment (pH 7.4 without GSH). In addition, pH sensitive DOX loaded micellar system (DOX/PAT) was prepared as a control. Furthermore, compared with DOX/PAT micelles, DOX/PCT micelles showed the stronger cytotoxicity against tumor cells to achieve an effective antitumor effect. After being internalized by clathrin/caveolin-mediated endocytosis and macropinocytosis, DOX/PCT micelles were depolymerized in intercellular acidic and a reductive environment to release DOX rapidly to kill tumor cells. Additionally, DOX/PCT micelles had a better inhibitory effect on tumor growth than DOX/PAT micelles in in vivo antitumor activity studies. Therefore, pH/reduction dual sensitive PCT polymers have great potential to be used as repaid release nanocarriers for intercellular delivery of antitumor drugs.

## 1. Introduction

Doxorubicin (DOX) is the most common chemotherapeutic drug for cancer treatment, while its deficiencies in physical properties, such as poor water solubility, short half-life in blood, and severe cardiac and renal toxicity during chemotherapy, limit the clinical application [1]. Although DOX can be delivered in some nanocarriers to avoid its side effect, the release behavior that can be controlled in the appropriate site remains a challenge. It is known that the surrounding environment of tumor tissues is quite different from that of normal physiological tissues in the process of tumor growth, such as changes in cell composition, including tumor cells, immune cells, fibroblasts, endothelial cells and pericytes, etc. In addition, there are changes in the concentration and types of cytokines secreted by the above-mentioned cells. This series of cells and related substances constitute a complex dynamic network around tumor tissues, which is named as tumor microenvironment [2]. Compared with the normal physiological environment, tumor microenvironment shows the characteristics of hypoxia, low pH, high tissue pressure, great difference between internal and external redox environment, and so on. These differences can promote the birth and development of tumor microenvironment responsive drug delivery system to solve the problem of non-selectivity of chemotherapeutic drugs for reducing side effects as well as improving bioavailability.

Due to the high modifiability of structure and nano-sized morphology, which makes it easier to accumulate in tumor tissues through enhanced permeability and retention (EPR) effect [3,4,5], polymeric micelles have been extensively applied in the tumor microenvironment responsive drug delivery system [6,7,8]. Under pathological state, the internal and external environment of the normal pH is greatly affected so that extracellular pH can be more acidic than normal physiological pH, which is due to the incomplete vascular system of tumor tissue and the resulting anaerobic environment [9]. In addition, there is a certain pH gradient in cytoplasm, endosome, and lysosome. Polymeric micelles with pH sensitivity can disintegrate to release the drugs at low pH value. The common pH sensitive structures are hydrazone bond [10], ketals [11], acetals [12], etc. Since 1980, both linear and crosslinked poly(ortho esters) (POE) as pH responsive polymers have been successfully investigated by Heller as biodegradable and biocompatible carrier for the drug delivery and POE hydrolysis mechanism in acid environment is autocatalytic degradation [13,14]. The polymer first hydrolyzes to the mono- and diesters. These mono- and diesters then hydrolyze further to acetic or propionic acids and the corresponding alcohols [15]. Hence, the cyclic orthoester group can be destroyed under the condition of low pH, which leads to the change in hydrophobicity of the whole substance. So, its application in polymeric micelles is beneficial to the disintegration of micelles in an acidic environment and the drugs release. Although the pH sensitive polymeric micelles containing orthoester can effectively release the drug in response to low pH in the tumor site, it is still difficult to release the drug completely and quickly only with the stimulation of low pH [16].

Except for the pH difference, the concentration of reduced glutathione (GSH) in tumor cells is approximately 100–1000 times higher than that in blood and normal tissues [17]. Therefore, the development of nanocarriers in response to GSH is considered to be a potential method for targeted delivery of anticancer drugs. The most common structure used in reduction responsive polymeric micelles is the disulfide bond (-S-S-) [18,19,20,21], which can be kept stable in plasma and broken in reduction conditions. The extracellular oxidation environment and intracellular reduction environment make it an important tool for the design of tumor microenvironment responsive polymeric micelles [22]. Low pH and reduction conditions, as the characteristics of tumor microenvironment, can be integrated to achieve a variety of effects, such as enhancement of the targeting of drug release in lysosomes and other internal chambers of tumor cells, the realization of rapid drug release into cytoplasm and nucleus, etc. [23,24,25,26,27,28,29,30,31,32].

**Scheme 1:**
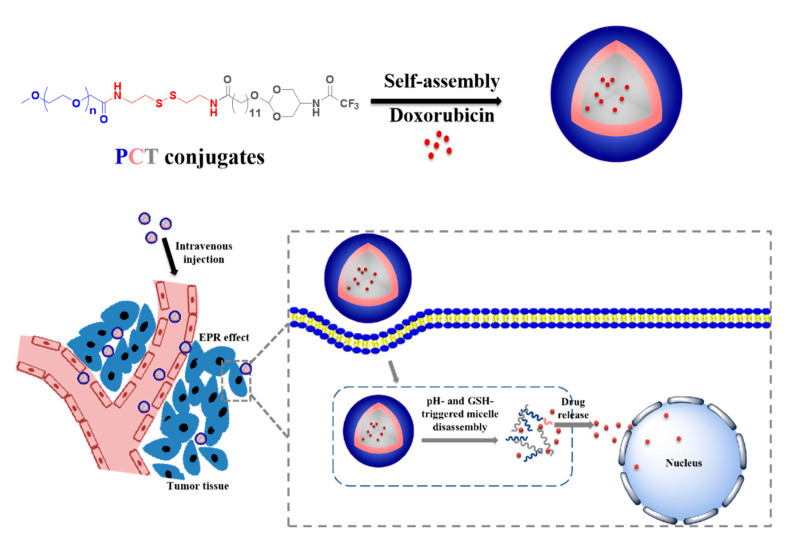
Schematic demonstration of preparation and anti-tumor effect of the pH/reduction sensitive DOX/PCT micelles.

To accelerate the drugs release in response to an appropriate stimulus such as temperature, pH, redox, reactive oxygen species, and enzymes, various dual sensitive polymeric micelles have been reported. Although some researchers have studied dual sensitive polymeric micelles, the impact of introducing disulfide bond into polymeric micelles containing orthoester group on its drug release and anti-tumor effect has not been investigated. Herein, a rapid-drug-release polymeric micelle system based on pH sensitive and reduction sensitive characteristics was constructed to effectively deliver DOX into tumor cells (Scheme 1). The novel dual sensitive polyethylene glycol–cystamine–12-hydroxydodecanoic acid–trifluoroacetamide orthoester (PCT) polymers containing disulfide bond and orthoester group were synthesized successfully, while pH single sensitive polyethylene glycol–adipic acid dihydrazide–12-hydroxydodecanoic acid–trifluoroacetamide orthoester (PAT) polymers were synthesized as a control. The responsive features, drug release behavior, cytotoxicity, cellular uptake, uptake mechanism, and subcellular localization of the micelles were explored thoroughly. Furthermore, the in vivo antitumor effect of the micelles was investigated by establishing B16 tumor-bearing nude mice model.

## 2. Material and Methods

### 2.1. Materials

Carboxylated polyethylene glycol (mPEG_2K_-COOH) was purchased from Shanghai Yare Biotechnology Co. Ltd. (Shanghai, China). Additionally, 1-Hydroxybenzotriazole (HOBT), 1-ethyl-3-(3-dimethylaminopropyl) carbodiimide (EDC), cystamine dihydrochloride, 12-hydroxydodecanoic acid (12-HDA), serinol, ethyl trifluoroacetate, trimethyl orthoformate and glutathione (GSH) were supplied by Aladdin Regent Co. (Shanghai, China). Doxorubicin hydrochloride was obtained from Beijing Huafeng United Technology Co. Ltd. (Beijing, China). N, N-Dimethylformamide (DMF), triethylamine (TEA), dichloromethane, petroleum ether, tetrahydrofuran (THF), methanol and ethyl acetate were purchased from Shanghai Reagent Chemical Co. (Shanghai, China). The dialysis bag (MWCO 1000) was purchased from Nantong haizhixing experimental equipment Co. Ltd. (Nantong, China). The 4′, 6-diamidino-2-phenylindole (DAPI) were supplied by Shanghai Beyotime Biotechnology Co. Ltd. (Suzhou, China). Fetal bovine serum (FBS), BASIC RPMI 1640 Medium, 3-(4,5-dimethylthiazol-2-yl)-2,5-diphenyl tetrazolium bromide (MTT), penicillin–streptomycin solution and LysoTracker Green were obtained from Life Technologies™ (Carlsbad, CA, USA). B16 mouse melanoma cells were purchased from the Chinese Academy of Sciences (Shanghai, China). The other chemicals were of analytical grade.

### 2.2. Synthesis and Characterization of PCT Polymers

#### 2.2.1. Synthesis of mPEG-CYS-(12-HDA)

**Figure 1:**
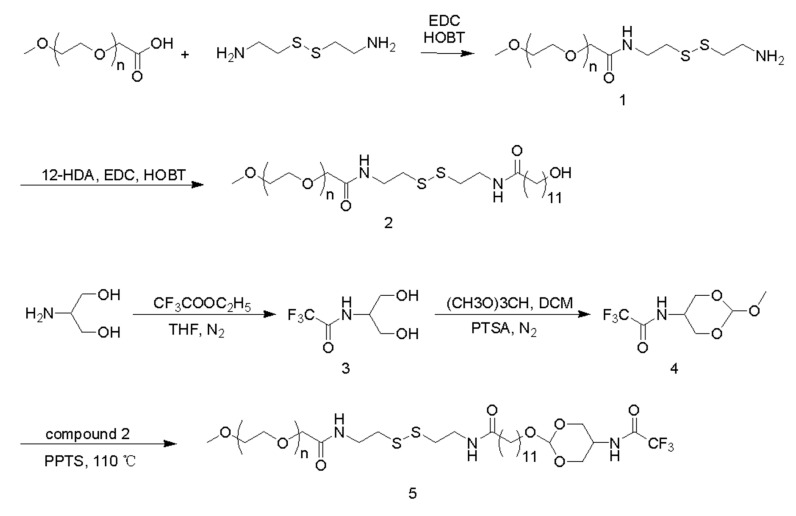
Synthesis route of PCT polymers.

mPEG_2K_-COOH (1 g, 0.5 mmol), HOBT (135 mg, 1 mmol), and EDC·HCl (192 mg, 1 mmol) were dissolved in 20 mL anhydrous DMF and activated for 4 h in an ice bath. Then, 10 mL DMF containing cystamine (304 mg, 2 mmol) was added to the above solution. Before the reaction, cystamine dihydrochloride was neutralized by sodium hydroxide in water and extracted with DCM. After stirring for 48 h, the reaction mixture was purified by dialysis for 3 days and freeze-dried to obtain mPEG_2K_- CYS (product 1 in Figure 1, yield approximately 90%). mPEG_2K_-CYS (300 mg, 0.15 mmol), 12-HAD (324.5 mg, 1.5 mmol), HOBT (405 mg, 3 mmol), and EDC·HCl (575 mg, 3 mmol) were added into 20 mL anhydrous DMF to react 48 h at room temperature. mPEG-CYS-(12-HDA) (product 2 in Figure 1, yield about 90%), which was confirmed by ^1^H NMR spectrometry (AVANCE III, 400 MHz, Bruker, Germany) in CDCl_3_ was obtained after dialysis and lyophilization.

#### 2.2.2. Synthesis of Trifluoroacetamide Orthoester (TDA)

TDA was prepared according to our previous report [33]. Firstly, a drip of ethyl trifluoroacetate (15.52 g, 110 mmol) was added into the serinol (9.11 g, 100 mmol) THF solution and kept in a 35 °C oil bath for 4 h under the protection of nitrogen. After removing the solvent by rotary evaporation, 60 mL EA was used to dissolve the product and then washed twice with 10 mL saturated sodium hydrogen sulfate solution and 10 mL saturated sodium chloride solution to remove impurities. The anhydrous magnesium sulfate was used to remove the water left in organic phase, and the solvent was concentrated to obtain the product 3 (Figure 1, yield about 60%).

Product 3 (3.57 g, 19.07 mmol), trimethyl orthoformate (9.71 g, 91.5 mmol), and p-toluenesulfonic acid (PTSA, 0.04 g, 0.23 mmol) were dissolved in DCM (46 mL) at 30 °C and stirred under nitrogen protection for 10 h. Then, triethylamine (300 μL) was added to terminate the reaction. After the solvent was removed, the product was dissolved by DCM (60 mL) and washed three times with 10 mL saturated sodium hydrogen carbonate solution and saturated sodium chloride solution, respectively. After washing, the organic phase was treated with anhydrous potassium carbonate. Silica gel column chromatography was used to purify the product with a mixture solvent of petroleum ether and EA (4:1) to obtain TDA (product 4 in Figure 1, yield approximately 45%), which was characterized by ^1^H NMR spectrometry in CDCl_3_.

#### 2.2.3. Synthesis of PCT Polymers

mPEG-CYS-(12-HDA) (314.8 mg, 0.0134 mmol), pyridinium p-toluene sulfonate (PPTS, 25 mg, 0.1 mmol), and TDA (306.8 mg, 1.34 mmol) were dissolved in 10 mL methylbenzene, refluxed 4 h at 110 °C and then purified with dialysis (yield about 85%). After lyophilization, PCT polymers were obtained and confirmed by ^1^H NMR spectrometry in CDCl_3_. The pH single sensitive polymer PAT polymers were synthesized by a similar method, in which adipic acid dihydrazide (ADH) was used to replace the cystamine (Figure S1).

### 2.3. Preparation and Characterization of the Micelles

The micelles of DOX/PCT and DOX/PAT were prepared by solvent evaporation method. Briefly, 10 mg of PCT and PAT polymers were dissolved in 10 mL PBS (0.01 M, pH 7.4), in which 2 mL DOX solution (1 mg/mL) was added under stirring. The mixture was stirred in the ventilation cabinet for 12 h to volatilize the tetrahydrofuran and then treated with a probe ultrasonic instrument (JY92-IIN, Ningbo Scientz Biotechnology Co., Ltd., Ningbo, China). The micelles were obtained successfully after filtration and ultrafiltration. A similar method was used to prepared PCT and PAT blank micelles.

The Zeta potential, particle size, and polydispersity index (PDI) was determined by dynamic light scattering method (DLS). Additionally, the morphology of DOX/PCT and DOX/PAT micelles was evaluated by transmission electron microscope (TEM, JEM-2100, Tokyo, Japan).

UV–vis spectrophotometer (UV-2550, Shimadzu, Kyoto, Japan) was used to observe the drug loading content (DL) and encapsulation efficiency (EE) of the micelles. Briefly, 500 μL drug-loaded micelles was fixed to 10 mL with formamide, and then the mixture went through ultrasonic treatment (200 W, 30 min) so that the structure of the micelles was completely destroyed. Then, DL and EE were tested at 480 nm.

### 2.4. pH and Reduction-Triggered Stability of the Micelles

The pH and reduction sensitivity of PCT blank micelles were evaluated using DLS. To prepare the 1 mg/mL solutions of PCT micelles containing different pH (5.0 and 7.4) and different GSH concentrations (0 and 10 mM), they were placed in a shaker (100 rpm, 37 ± 0.5 °C). After 48 h, the particle size distributions of PCT micelles under different conditions were measured by DLS.

### 2.5. In Vitro Drug Release Study

DOX, released from DOX/PCT and DOX/PAT micelles under different pH and GSH concentrations, which simulate the normal physiological environment and tumor microenvironment in vivo, was investigated by dynamic dialysis method; DOX/PCT and DOX/PAT micelles (2.0 mL) were dialyzed in deionized water at room temperature and immersed in PBS (0.01 mol/L, 20 mL) with different pH (pH 7.4 and pH 5.0) and different GSH concentrations (0 and 10 mM) [34]. Then, 1 mL was taken from the PBS outside the dialysis bag at different time points (0.5, 1, 2, 3, 4, 6, 8, 10, 12, 24, 36, 48, 72 h) for measurement, and new PBS was added in order for the volume of the release medium to stay the same. A UV–vis spectrophotometer was chosen to evaluate the release behavior of DOX from DOX/PCT and DOX/PAT micelles.

### 2.6. Cytotoxicity Test

B16 cells (5 × 10^3^ cells/well) were cultured in 96-well plates for 12 h (37 °C, 5% CO_2_) for adherence. The original medium was replaced by blank micelles (10−200 mg/mL), DOX/PCT, DOX/PAT micelles and free DOX (the concentrations ranged from 0.01 to 5 μg/mL) for incubation another 48 h. After removing the medium and incubating with the MTT solution (0.5 mg/mL, 100 μL) for 4 h, the MTT solution was replaced by DMSO (100 μL) to dissolve the blue–violet crystals. After the crystals were completely dissolved, the absorbance was measures by a microplate reader (Multiskan MK3, Thermo, Waltham, MA, USA).

### 2.7. Cellular Uptake

The cellular uptake of DOX/PAT and DOX/PCT micelles was observed qualitatively by inverted fluorescence microscope (IFM). B16 cells were inoculated in 6-well plates (1.5 × 10^5^/well) and cultured for 12 h. Then, the original culture medium was replaced by 2 mL medium-diluted free DOX, DOX/PAT and DOX/PCT (amount to 5 μg/mL DOX) for different times. After that, the cells were gently washed with PBS. Then, the cells were fixed with paraformaldehyde solution (4%, 200 μL, 20 min) and washed with PBS, then incubated with DAPI (200 μL) for 20 min to visualize the nucleus, and finally, washed with PBS again. Finally, an inverted fluorescence microscope (Leica, DMIL, Wetzlar, Germany) was used to observe the cellular uptake of the micelles after adding PBS (200 μL/well) into the plates.

Furthermore, flow cytometry was chosen to quantify the cellular uptake of DOX/PAT and DOX/PCT micelles. B16 cells (2 × 10^5^ cells/well) were cultured in 6-well plates for 12 h. After discarding the original culture medium, the cells were co-incubated with 2 mL medium-diluted DOX/PAT micelle solution, DOX/PCT micelle solution and free DOX solution (DOX concentration was 5 μg/mL) for different times, respectively, and then washed with PBS. The cells were collected and then dispersed in PBS (500 μL/well) and determined by flow cytometry (Becton Dickinson FACSCalibur, Franklin Lakes, NJ, USA).

### 2.8. Cellular Uptake Mechanism of the DOX/PCT Micelles

Flow cytometer was also used to measure the uptake mechanism of DOX/PCT micelles by B16 cells. Firstly, B16 cells cultured for 12 h in 6-well plates (2 × 10^5^/well) were incubated at 4 °C for 1 h to test the effect of low temperature on cell uptake; then, DOX/PCT micelles (5 μg/mL DOX) were added for 1 h incubation. Secondly, in order to explore the cellular uptake pathway of DOX/PCT micelles by B16 cells, B16 cells were inoculated in 6-well plates (2 × 10^5^/well) and cultured for 12 h. Then, the original culture medium was replaced by various inhibitors diluted by the culture medium (1 µg/mL Indomethacin [35], 10 µg/mL Chlorpromazine [36], 40 µg/mL Colchicine [37], 200 µM Genistein) [38]. After 1 h, the mixed solution containing DOX/PCT micelles (DOX concentration was 5 μg/mL) and above-mentioned inhibitors were used to replace the former solution and incubated with cells for 1 h. At the end of culture, all the cells were washed and collected for the test of flow cytometry. The control group was treated with DOX/PCT micelle solution (DOX concentration was 5 μg/mL) for the same time, and its fluorescence intensity was set as 100%. In order to eliminate the effect of the toxicity of inhibitors on cell uptake, the cytotoxicity of the inhibitors of predescribed concentration to B16 cells was tested in advance.

### 2.9. Subcellular Localization of the Micelles

Confocal laser scanning microscope (CLSM, Leica, TCS SP8, Germany) was used to measure the intracellular transport of DOX/PCT micelles. B16 cells were cultured for 12 h on a cell culture dish (φ 15 mm) at 1.5 × 10^5^ cells/dish. The old culture medium with DOX/PCT micelles (amount to 5 μg/mL DOX) was replaced, and the cells were incubated for a certain time (1 h, 2 h and 4 h). After washing the cells for several times with PBS, 200 μL LysoTracker Green (50 nM) was added to dye the lysosomes. After removing LysoTracker Green, the cells were washed again and fixed with paraformaldehyde solution (4%, 200 μL) for 20 min. The cells were further stained 20 min by 300 μL DAPI to visualize the nuclei. After being washed by PBS, the cells were observed under CLSM in 200 μL PBS.

### 2.10. In Vivo Anti-Tumor Study

This study was approved by the Ethical Approval for Research Involving Animals of Jiangnan University (JN. No 20190630c0500970, 30 June 2019). The four week-old female BALB/c nude mice were loaded with tumors by abdominal subcutaneous inoculation of B16 cell suspension (5 × 10^6^ cells/mouse). The mice were used for the study of the anti-tumor activity in vivo of the drug-loaded micelles. The diameter of the tumor was recorded daily after the injection to calculate the tumor volume, and the formulations were administrated when tumor volume grew to approximately 50 to 100 mm^3^.

Four groups were randomly assigned according to the tumor volume of nude mice. Free DOX, DOX/PAT, and DOX/PCT solutions were injected into the nude mice (10 mg DOX/kg) on days 1, 3, and 5, respectively, using the corresponding volume of saline as a control. After administration, the growth state of nude mice was closely observed, and the body weight of the nude mice was measured every day. The length and width of each nude mouse tumor in each group were measured by Vernier caliper. On the 10th day after administration, all nude mice were sacrificed, and the subcutaneous tumors were carefully separated, weighed and fixed with 4% paraformaldehyde to perform hematoxylin and eosin (H&E) staining.

### 2.11. Statistical Analysis

All experiments were repeated in three times and data were presented as the means ± SD. One-way ANOVA (SPSS 17.0, Chicago, IL, USA) was applied to statistical analysis. A value of *p <* 0.05 was considered significant.

## 3. Results and Discussion

### 3.1. Synthesis and Characterization of the Polymers

**Figure 2:**
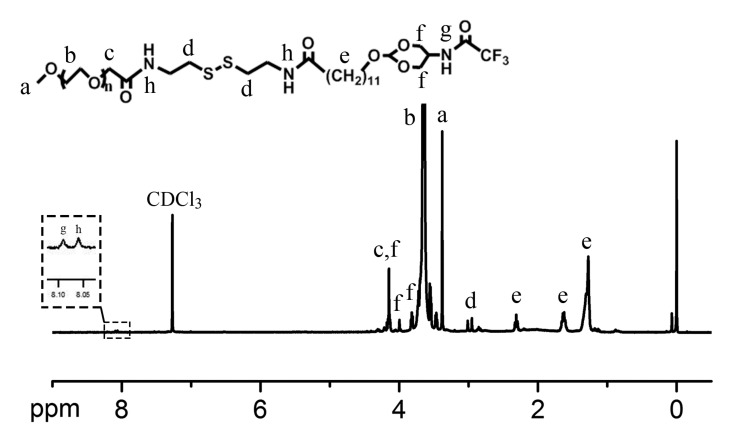
^1^H NMR spectra of PCT polymers in *CDCl_3_*.

The synthesis routes of PCT and PAT polymers were shown in Figure 1 and Figure S1, respectively. The PEG was linked with 12-HDA through the amino group of cystamine, and then the product reacted with TDA to finally obtain PCT polymers. The ^1^H NMR spectrum of PCT polymers was shown in Figure 2. The typical peaks of methylene and terminal methyl of PEG were 3.63 and 3.37 ppm, respectively, and the methylene in CYS showed at 2.75−3.06 ppm. Moreover, 1.27, 1.63, and 2.31 ppm were assigned to the methylene of 12-HDA, while the methylene in TDA appeared at 3.80, 3.99, and 4.14 ppm. The above results proved that PCT polymers were synthesized successfully. The ^1^H NMR spectrum of intermediate products (TDA, mPEG-CYS, and mPEG-CYS-12-HAD) and PAT polymers were shown from Figures S2–S5 in Supporting Information.

### 3.2. Formation and Characterization of Blank and DOX-Loaded Micelles

**Figure 3:**
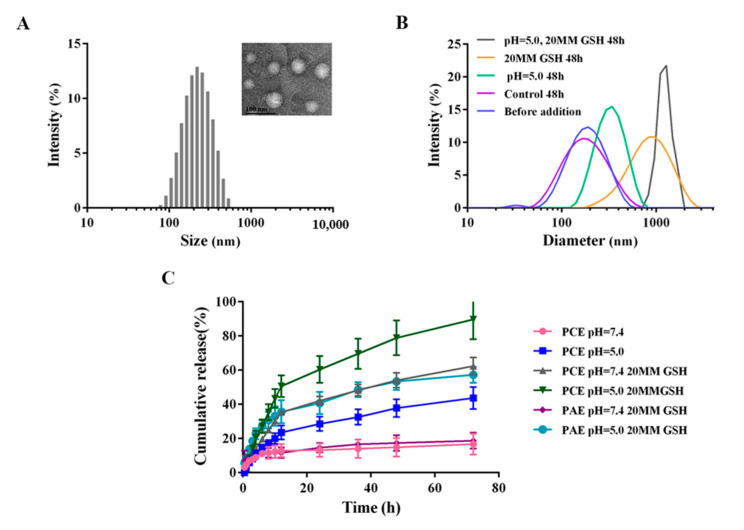
(**A**) The size distribution and morphology of DOX/PCT micelles. (**B**) The particle size changes and (**C**) in vitro cumulative drug release behaviors of the micelles in different pH/GSH condition. Data were expressed as mean ± SD (*n* = 3).

The particle size and zeta potential of blank and DOX-loaded micelles are shown in Table 1. The particle size of the blank micelles formed by PAT and PCT polymers was approximately 180 nm, while it increased to ~200 nm after loading DOX, which may be due to the fact that the internal drugs occupy more space. All the micelles showed slight negativity, which was beneficial for the micelles to avoid the adsorption of plasma proteins and deliver more drugs to the tumor site. Moreover, both DOX/PCT (Figure 3A) and DOX/PAT micelles (Figure S6) have regular spherical structure with uniform particle size distribution. In addition, the EE and DL of DOX-loaded micelles were above 85% and 10%, respectively, which suggests that PCT and PAT polymers had the same drug loading capacity resulting from similar chemical structure.

### 3.3. pH and Reduction-Triggered Stability of the Micelles

The particle size changes of PCT micelles under different GSH and pH conditions were measured by DLS at 48 h to verify the pH and reduction sensitivity. In theory, PCT have an orthoester group which is a pH sensitive group. When the particles were in solution of pH 5.4, the orthoester groups would break, and resulted in the expansion the particles. The particle size was almost unchanged under pH 7.4 without GSH condition (Figure 3B), while it increased to about 350 nm when the pH decreased to 5.0. When the condition changed to pH 7.4 and 10 mM GSH, the particle size increased to about 900 nm. Most obviously, the micelles swelled to over 1000 nm under pH 5.4 and 10 mM GSH condition. The results showed that both a high concentration of GSH and a low pH environment could lead to an obvious change in the micellar structure, which is consistent with the theory, implying that PCT micelle had pH and reduction sensitivity.

### 3.4. In Vitro Drug Release Study

The drug release behavior of DOX/PCT and DOX/PAT micelles in PBS with different pH and GSH was investigated by dynamic dialysis method. As shown in Figure 3C, the cumulative release of DOX/PCT micelles in pH 5.0/0 mM GSH PBS at 72 h was 2.62 times higher than that in pH 7.4/0 mM GSH PBS (*p* < 0.05), which might be that the orthoester group in PCT was hydrolyzed under acidic conditions to accelerate drug release. Similarly, DOX/PAT micelles with orthoester group also showed pH sensitive release behavior (*p* < 0.01) from Figure 3C. The cumulative release of DOX/PCT micelles in pH 7.4/10 mM GSH PBS was 62.4 ± 5.1%, which was 3.74 times higher than that in pH 7.4/0 mM GSH PBS (*p* < 0.001). The reason was that the disulfide bond in PCT was broken at high GSH concentration, causing the micellar structure to be destroyed and drug to be released. Furthermore, the highest cumulative release amount of DOX/PCT micelles was observed in pH 5.0/10 mM GSH PBS, which was 1.56 times higher than that of DOX/PAT micelles (*p* < 0.01). Compared with pH sensitive PAT micelles, pH/reduction dual sensitive PCT micelles can be depolymerized more easily under the condition of low pH/high GSH concentration, leading to the faster release rate of DOX. These results suggested that the drugs were released from DOX/PCT micelles slowly in normal physiological environment, but when the micelles reached tumor microenvironment, drugs were released quickly due to the structural destruction to achieve the tumor killing effects.

### 3.5. Cytotoxicity Evaluation

**Figure 4:**
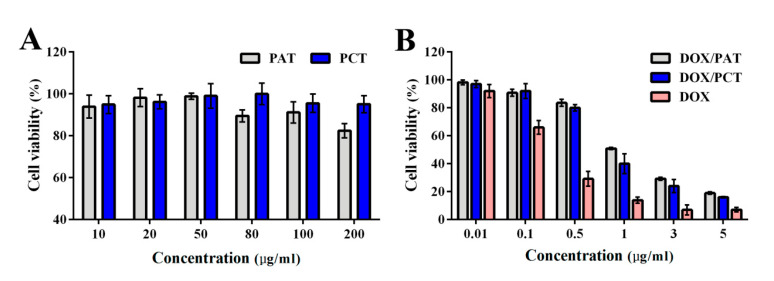
(**A**) Cytotoxicity of PAT and PCT micelles with different concentration, (**B**) cytotoxicity of DOX-loaded micelles and free DOX micelles with different concentration (mean ± SD *n* = 3).

The in vitro cytotoxicity of PCT, PAT, DOX/PAT, and DOX/PCT micelles was evaluated in B16 cells. As shown from Figure 4A, the viability of B16 cells was more than 85% at all concentrations of the polymers, indicating that both PAT and PCT had no obvious toxicity and can be used as drug carriers. As shown in Figure 4B, the cytotoxicity of DOX against B16 cells was dose-dependent, which indicated that the cytotoxicity of DOX depended on the concentration. Moreover, the half-maximal inhibitory concentration (IC_50_) of DOX/PAT (0.856 ± 0.011) and DOX/PCT (0.570 ± 0.026) micelles were significantly higher than that of free DOX (0.185 ± 0.037) (*p* < 0.01), which might be that free DOX as a small molecule was internalized by tumor cells more easily than that of micelles. However, the IC_50_ value of DOX/PCT was lower than that of DOX/PAT (*p* < 0.05), showing higher cytotoxicity. After being uptaken into the tumor cells, the micelles can encounter acid and reduction environment, so dual sensitive DOX/PCT micelles can be depolymerized to release DOX quickly to kill tumor cells more effectively than single sensitive DOX/PAT micelles.

### 3.6. Cellular Uptake

**Figure 5:**
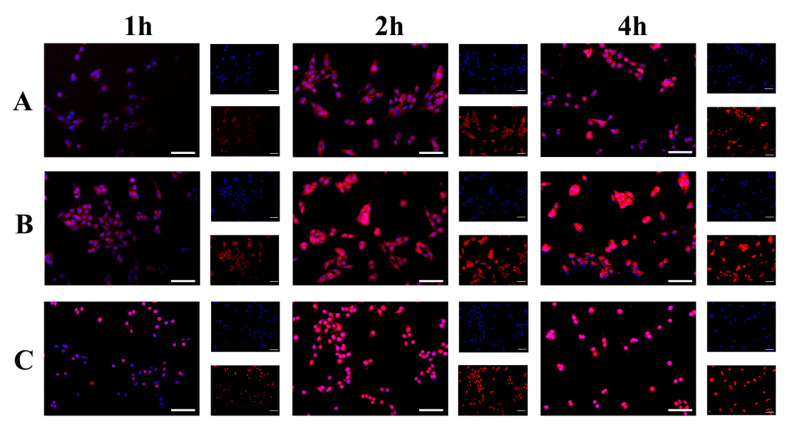
Fluorescence microscopy images of B16 cells incubated with (**A**) DOX/PAT, (**B**) DOX/PCT and (**C**) free DOX. Blue and red colors indicate nucleus and DOX, respectively (The bar is 200 μm).

**Figure 6:**
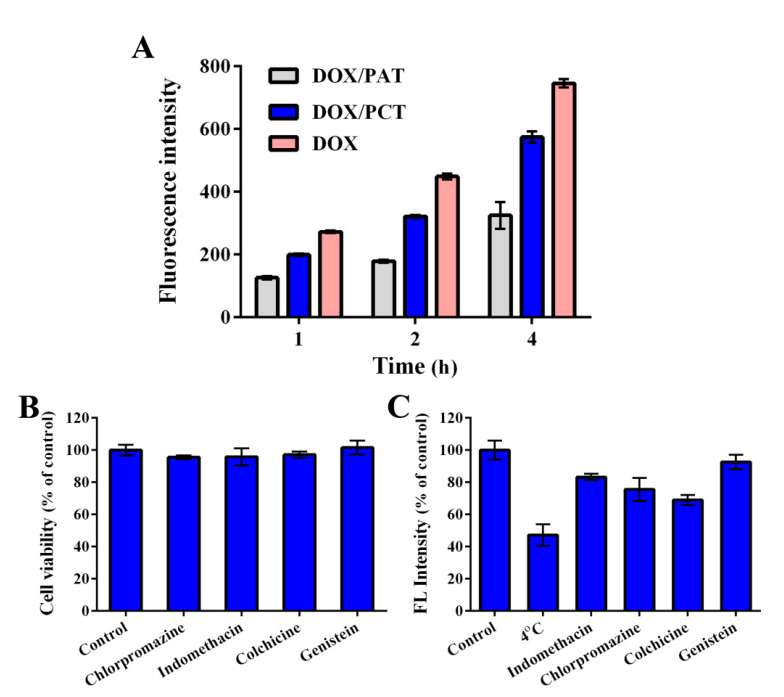
(**A**) The uptake of free DOX and DOX-loaded micelles at 1, 2, and 4 h in B16 cells. (**B**) Cytotoxicity of different endocytosis inhibitors and (**C**) the effects on the uptake of DOX/PCT of different endocytosis inhibitors (mean ± SD (*n* = 3)).

Cellular uptake of free DOX and drug-loaded micelles was observed qualitatively by fluorescence microscope after B16 cells were co-cultured with free DOX, DOX/PAT, and DOX/PCT micelles for 1, 2, and 4 h, respectively. In Figure 5, the fluorescence intensity of DOX increased with time in all groups, indicating that cellular uptake of both free DOX and micellar groups was time-dependent. Additionally, free DOX entered the cancer cells and nucleus faster, which was shown to higher fluorescence intensity than drug-loaded micelles, thus free DOX achieving stronger tumor cytotoxicity. As expected, the intracellular fluorescence intensity of DOX/PCT micelles was always stronger than that of DOX/PAT micelles. In addition, the uptake of the micelles in B16 cells was further studied quantitatively by flow cytometry. As shown in Figure 6A, the fluorescence intensity of free DOX was still highly significantly higher than that of DOX-loaded micelles (*p* < 0.01). However, the fluorescence intensity of DOX/PCT micelle group was significantly higher than that of DOX/PAT micelles (*p* < 0.001) at 4 h. These results were caused by the fast release of DOX in pH/reduction tumor microenvironment. Combined with cytotoxicity, these results showed that DOX/PCT micelles had better drug release efficiency in cancer cells to achieve the better effect of killing cancer cells.

### 3.7. Cellular Uptake Mechanism of DOX/PCT Micelles

Generally speaking, there are two kinds of uptake pathways: endocytosis pathway and non-endocytosis pathway [39]. Polymeric micelles are mainly uptaken by cells through endocytosis pathway because of its size distribution [40]. The endocytosis pathway is divided into four main pathways: clathrin-mediated endocytosis, of which chlorpromazine is usually used as inhibitor; caveolae-mediated endocytosis, of which indomethacin is usually used as inhibitor; macropinocytosis, of which colchicine is usually used as inhibitor; and clathrin/caveolae-independent endocytosis, of which genistein is usually used as inhibitor [41]. The cellular uptake mechanism of DOX/PCT micelles was explored using various inhibitors. First, the survival rate of B16 cells treated with the selected concentration of inhibitors for 48 h was more than 90% (Figure 6B), indicating that they had no toxicity to tumor cells and does not affect the uptake of drug-loaded micelles in cancer cells through their own cytotoxicity. Additionally, as shown in Figure 6C, the uptake of DOX/PCT micelles decreased to (47.2 ± 6.6)% at low temperature compared with the control group (*p* < 0.01), indicating that energy was required during the uptake of DOX/PCT micelles by tumor cells. The cellular uptake of the control group and genistein group had no significant difference, indicating that clathrin/caveolae-independent endocytosis was not related to the uptake of DOX/PCT micelles. However, when the cells were treated with indomethacin, chlorpromazine, and colchicine, the cellular uptake of DOX/PCT micelles decreased to 83.2 ± 2.0, 75.5 ± 7.2, and 69.1 ± 3.1%, respectively (*p* < 0.05). The result indicated that multiple endocytosis pathways were involved in the uptake of DOX/PCT micelles by tumor cells, which contained clathrin-mediated endocytosis, caveolae-mediated endocytosis, and micropinocytosis.

### 3.8. Subcellular Localization of the Micelles

**Figure 7:**
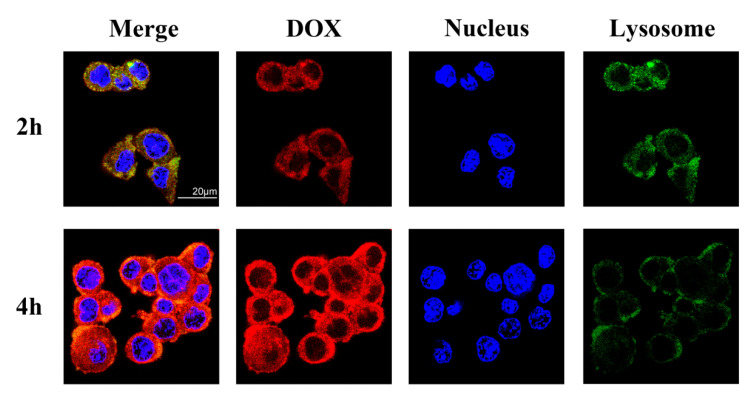
Confocal images of DOC/PCT in B16 cells for 2 h and 4 h. Blue, green, and red colors indicate nucleus, lysosome, and DOX, respectively.

After being uptaken by cancer cells, the micelles enter the lysosomes (acidic environment), so it is an extremely important issue that the micelles or drugs can escape into the cytoplasm and reach nucleus. Therefore, CLSM was used to observe the intracellular drug release and transport after the tumor cells were treated with DOX/PCT micelles for different times. As shown in Figure 7, after B16 cells were cultured with DOX/PCT micelles for 2 h, red fluorescence of DOX merged with the green fluorescence of lysosomes in B16 cells, exhibiting bright yellow fluorescence, which indicated that the DOX/PCT micelles reached the lysosomes. However, the yellow fluorescence intensity of the merged picture decreased after 4 h, while the red fluorescence in the cytoplasm increased. It indicated that the DOX/PCT micelles were depolymerized in the acidic environment and DOX were released and escaped into the cytoplasm and nucleus to achieve the anti-tumor effect.

### 3.9. In Vivo Anti-Tumor Study

**Figure 8:**
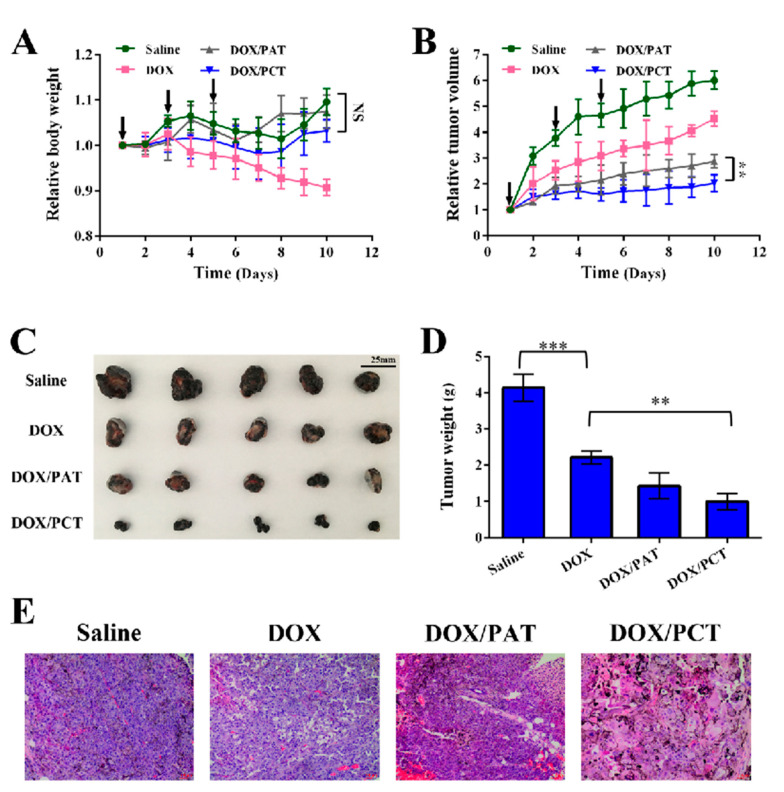
(**A**) The changes in body weight and (**B**) tumor volume of saline-, free DOX-, DOX/PAT-, and DOX/PCT-injected B16 tumor-bearing mice. (**C**) The pictures of excised tumor tissues and (**D**) tumor weight of different groups from mice after being sacrificed (mean ± SD, *n* = 5). (**E**) H&E staining results of tumors in different groups of BALB/c nude mice, (The bar is 40 μm). NS means *p* > 0.05 (not significant), ** means *p* < 0.01 (moderately significant) and means *** *p* < 0.001 (highly significant).

To investigate the antitumor effect of DOX/PCT micelles in vivo, B16 cells were inoculated to the nude mice subcutaneously in the abdomen. When the tumor reached to a certain size, the mice were injected with saline, free DOX, DOX/PAT, or DOX/PCT solution through tail vein, respectively. The safety of each group and the inhibitory effect on tumor growth were evaluated by measuring the changes of body weight and tumor volume. As shown in Figure 8A, the body weights of mice of different groups are compared: the group of saline and DOX-loaded micelles have no significant difference, but the DOX group was significantly lower than other three groups (*p* < 0.05) at the end of the experiment. The results suggested that free DOX caused obvious side effects, while DOX-loaded micelles could reduce the toxicity to nude mice. Interestingly, though all the tumors were slowed down in free DOX, DOX/PCT micelles, and DOX/PAT micelles groups (Figure 8B), the micelles showed the stronger inhibitory effect than free DOX (*p* < 0.001). Although free DOX had the better anti-tumor effect in in vitro cytological evaluation, it would be diffused to the whole body, resulting in an unsatisfactory therapeutic effect and some side effects. However, DOX-loaded micelles with nanosized uniform distribution could be more easily accumulated in tumor region by EPR effect to achieve the better anti-tumor effect. Furthermore, the inhibitory effect of DOX/PCT micelles on tumor growth was more obvious than that of DOX/PAT micelles (*p* < 0.01), which resulted from the fast drug release rate in the special tumor environment.

The final tumor images of each group are shown in Figure 8C. The result also suggested that the DOX/PCT micelles had the strongest inhibitory effect on tumor growth. As shown in Figure 8D, the tumor weights of free DOX, DOX/PAT, and DOX/PCT micelles groups were significantly smaller than that of saline group (*p* < 0.001). Additionally, the tumor weights of the DOX/PCT group were the smallest, indicating that it had the strongest antitumor effect among the formulations (*p* < 0.01). Additionally, the treatment effect of DOX/PCT micelles was further evaluated by histological analysis tumor tissue sections (Figure 8E). However, the results of cytotoxicity in vitro showed that free DOX was more toxic than DOX-loaded micelles to cancer cells. The conflicts between the results in vitro and in vivo were probably because that free DOX could not accumulate in the tumor site during systemic circulation and was easily cleared by plasma protein resulting in short half-life, while the drug-loaded micelles can accumulate in the tumor through the EPR effect, and the low pH and reduction environment of the tumor microenvironment can promote the rapid release of drugs by DOX/PCT micelles to present the optimal tumor inhibitory effect.

## 4. Conclusions

The novel pH/reduction dual sensitive PCT polymers based on disulfide bond and cyclic orthoester groups were successfully synthesized in this study. PCT polymers could self-assemble and encapsulate DOX to form micelles with uniform particle size. The results of stability and drug release behavior under different pH/GSH conditions proved the pH and reduction sensitivity character of PCT micelles, which was in keeping with the plan as programmed. In in vitro cytotoxicity experiments, DOX/PCT micelles could be internalized into B16 cells through multi-path endocytosis and rapidly disintegrated to release drugs in the low pH and reductive environment, so that DOX could reach the nucleus to achieve a better anti-tumor effect than DOX/PAT micelles. Importantly, DOX/PCT micelles showed the strongest anti-tumor activity in vivo than free DOX and DOX/PAT micelles. Therefore, pH/reduction dual sensitive PCT polymers could enhance intracellular drug release and be used as nanocarriers for efficient delivery of hydrophobic anticancer drugs.

## Figures and Tables

**Table 1:** Characterization of blank and DOX-loaded PCT and PAT micelles. Data were expressed as mean ± SD (*n* = 3).

	Size (nm)	PDI	*Zeta* Potential(mV)	EE (%)	DL (%)
PAT	175.9 ± 8.3	0.221 ± 0.010	−4.512 ± 0.267	-	-
DOX/PAT	202.6 ± 7.1	0.123 ± 0.022	−2.271 ± 0.225	87.2 ± 1.67	12.1 ± 0.36
PCT	182.5 ± 10.7	0.206 ± 0.027	−3.228 ± 0.194	-	-
DOX/PCT	214.2 ± 9.8	0.182 ± 0.034	−2.390 ± 0.305	85.8 ± 2.65	11.4 ± 1.74

## Supplementary Materials

The following are available online at https://www.mdpi.com/article/10.3390/pharmaceutics13091464/s1, Supporting Information: The synthesis of mPEG-ADH-(12-HDA), TDA, and PTA polymer; H NMR spectra of TDA, mPEG-CYS, mPEG-CYS-(12-HDA), and PTA polymer in CDCl3; the size distribution and TEM micrograph of DOX/PAT micelles. Figure S1: Synthesis route of PAT polymer, Figure S2: ^1^H NMR spectra of TDA in CDCl3, Figure S3: 1H NMR spectra of mPEG-CYS in CDCl3, Figure S4: 1H NMR spectra of mPEG-CYS-(12-HDA) in CDCl3, Figure S5: 1H NMR spectra of PAT polymer in CDCl3, Figure S6: (A) The size distribution and (B) TEM micrograph of DOX/PAT micelles.

## Abbreviation

PCT	polyethylene glycol-cystamine-12-hydroxydodecanoic acid-trifluoroacetamide orthoester
PAT	polyethylene glycol-adipic acid dihydrazide-12-hydroxydodecanoic acid-trifluoroacetamide orthoester
TDA	trifluoroacetamide orthoester
ADH	adipic acid dihydrazide
EE	encapsulation efficiency
DL	drug loading content
