# Supplementary material for: Fabrication of pH/Reduction Sensitive Polyethylene Glycol-Based Micelles for Enhanced Intracellular Drug Release
Source: Pharmaceutics. 2021 Sep 14;13(9):1464. doi: 10.3390/pharmaceutics13091464 (PMC8470983; doi:10.3390/pharmaceutics13091464)
Supplement: Supplementary file 1 [file pharmaceutics-13-01464-s001.zip › pharmaceutics-1360075-supplementary.pdf]

pharmaceutics

MDPI

Supplementary Materials

Fabrication of pH/Reduction Sensitive Polyethylene Glycol-Based Micelles for Enhanced Intracellular Drug Release

Yang Yang, Fuwei Yang, Xiaotian Shan, Jiamin Xu, Wenjie Fang, Juan Zhou, Lipeng Qiu and Jinghua Chen

Synthesis of PAT polymers

The synthesis route of PAT polymers was shown in Figure S1.

Synthesis of mPEG-ADH-(12-HDA)

$mPEG_{2K}$-COOH (200 mg，0.1 mmol), adipic acid dihydrazide (ADH, 87.1 mg, 0.5 mmol) were dissolved in water (50 mL), in which EDC·HCl (38.3 mg, 0.2 mmol) and NHS (23.02 mg, 0.2 mmol) were added. The pH of the solution was adjusted to 4.75 with hydrochloric acid solution (0.1 mmol), and after 1 h of reaction, the pH of the solution was adjusted to 7 with sodium hydroxide solution (0.1 mmol). The mixture was purified by dialysis, and then lyophilized to obtain $mPEG_{2K}$-ADH (product 1 in Figure S1，yield about 90%).

$mPEG_{2K}$- ADH (400mg, 0.185mmol) in 10ml anhydrous DMF was added into 20 mL anhydrous DMF containing 12-HDA (399.6 mg, 1.85 mmol), EDC·HCl (575mg, 3mmol) and HOBT (405mg, 3mmol).The reaction was completed after the mixture was stirred at room temperature for 48 hours. As a result of dialysis and lyophilization, mPEG-ADH-(12-HDA) (product 2 in Fig. S1, yield about 90%) was obtained and confirmed by ^{1}H NMR spectrometry (AVANCE III, 400 MHz, Bruker, Germany) in $CDCl_3$.

Synthesis of 2, 2, 2-trifluoro-N-(2-methoxy-1, 3-dioxan-5-yl)-Acetamide (TDA)

The synthesis of TDA was the same as described in section 2.2.2 of the manuscript.

Synthesis of PAT polymers

mPEG-ADH-(12-HDA) (194.2 mg, 0.067 mmol), TDA (153.4 mg, 0.67 mmol), pyridinium p-toluene sulfonate (PPTS, 12.5 mg, 0.05 mmol) were dissolved in 10 ml methylbenzene, which was refluxed at 110 °C for 4 h and then purified through dialysis. After lyophilization, PAT polymers were finally obtained (yield about 85%). The structure of PAT polymers was characterized by ^{1}H NMR spectrometry in $CDCl_3$.

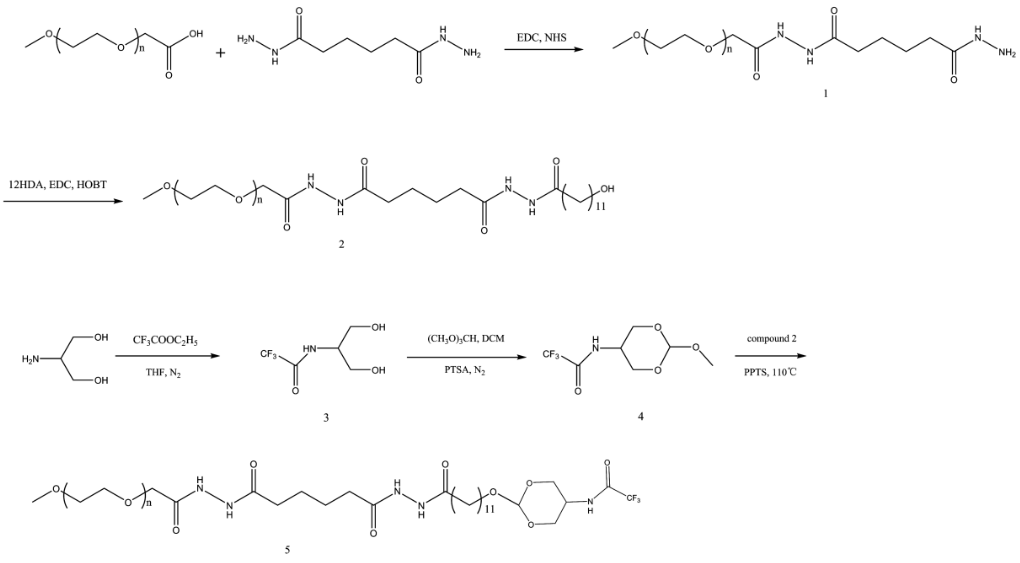

Figure S1. Synthesis route of PAT polymer.

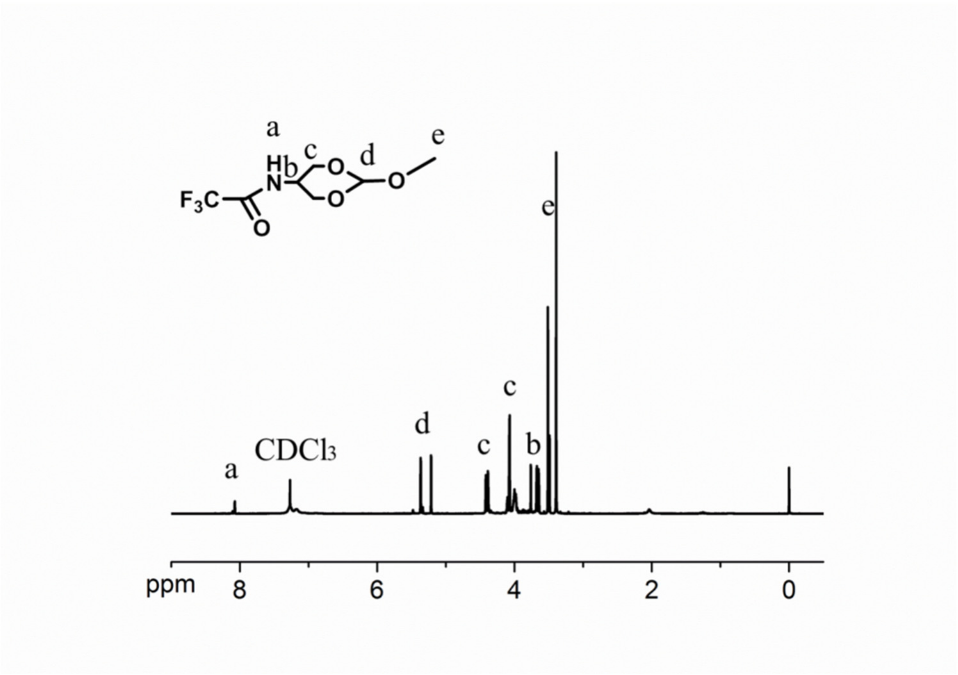

Figure S2. ^{1}H NMR spectra of TDA in $CDCl_3$.

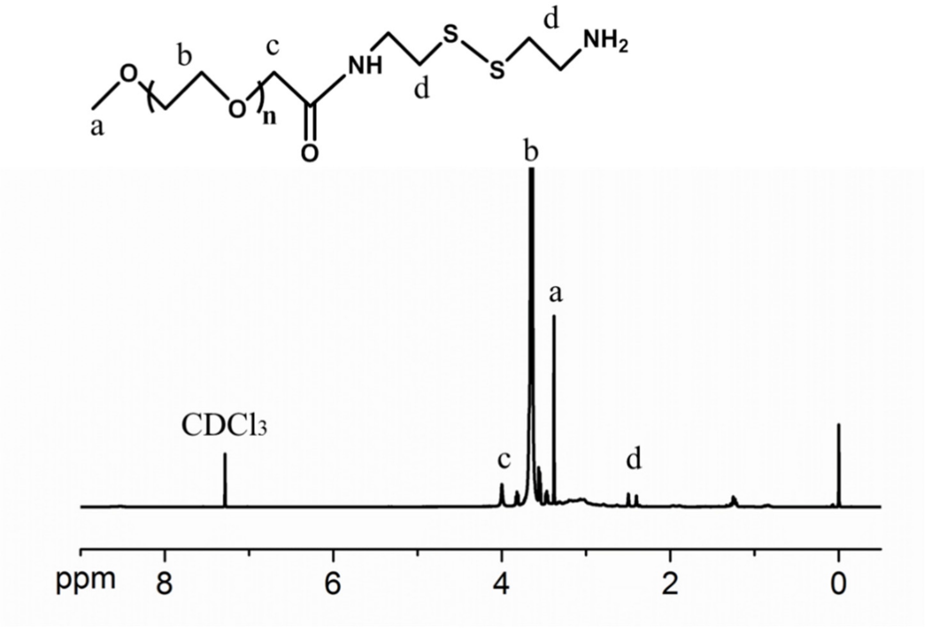

Figure S3. ^{1}H NMR spectra of mPEG-CYS in $CDCl_3$.

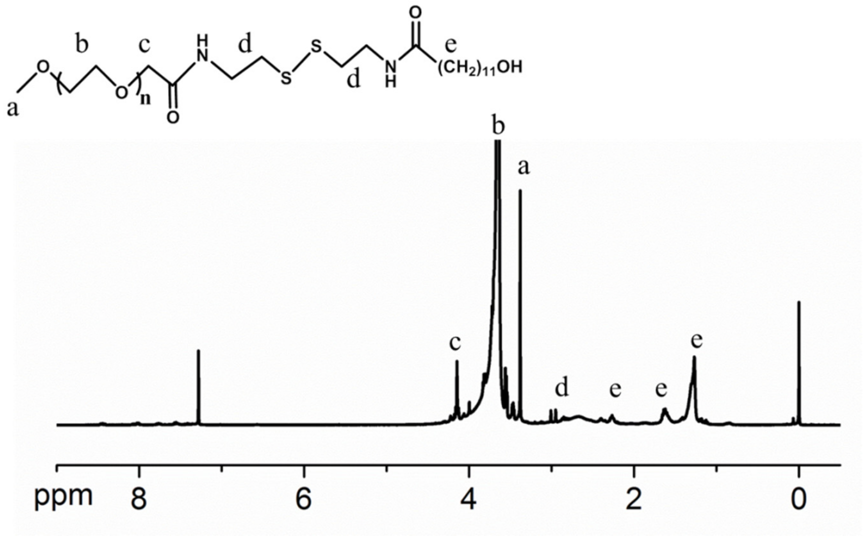

Figure S4. ^{1}H NMR spectra of mPEG-CYS-(12-HDA) in $CDCl_3$.

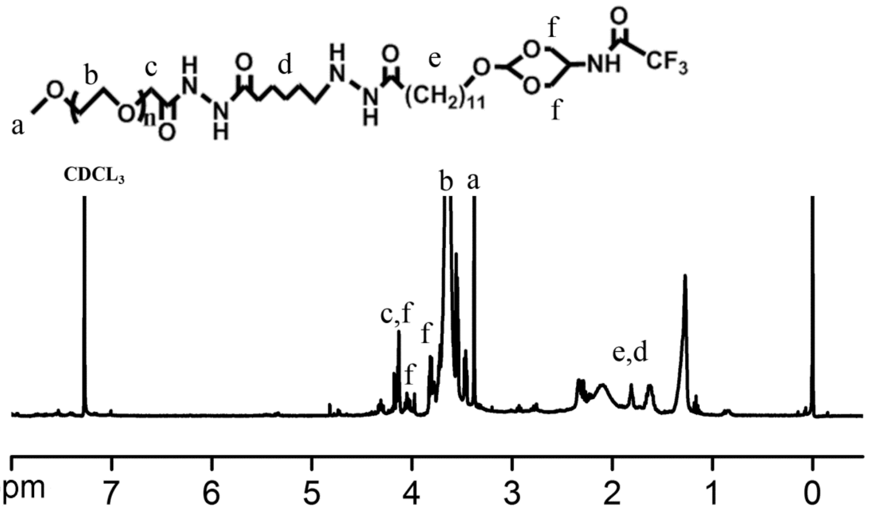

Figure S5. ^{1}H NMR spectra of PAT polymer in $CDCl_3$.

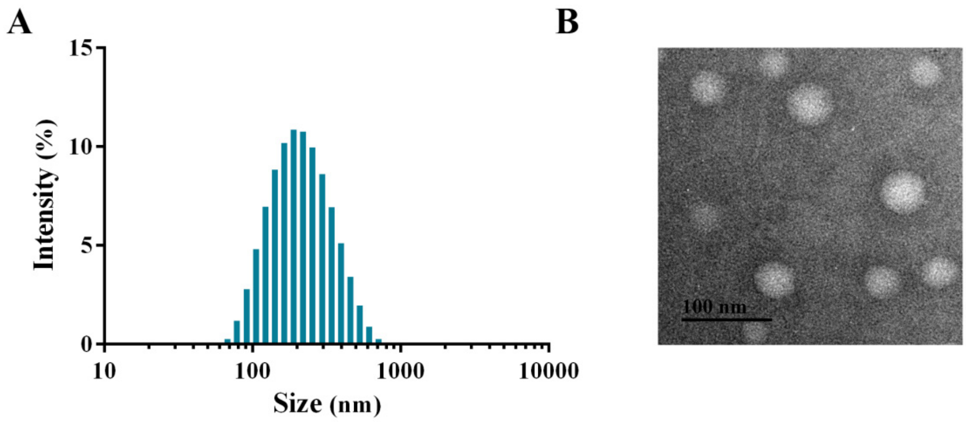

Figure S6. (A) The size distribution and (B) TEM micrograph of DOX/PAT micelles.
